# Reference Ranges for Uterine Artery Pulsatility Index during the Menstrual Cycle: A Cross-Sectional Study

**DOI:** 10.1371/journal.pone.0119103

**Published:** 2015-03-05

**Authors:** Luís Guedes-Martins, Rita Gaio, Joaquim Saraiva, Sofia Cerdeira, Liliana Matos, Elisabete Silva, Filipe Macedo, Henrique Almeida

**Affiliations:** 1 Department of Experimental Biology, Faculty of Medicine, University of Porto, 4200–319 Porto, Portugal; 2 IBMC-Instituto de Biologia Molecular e Celular, 4150–180 Porto, Portugal; 3 Centro Hospitalar do Porto EPE, Departamento da Mulher e da Medicina Reprodutiva, Largo Prof. Abel Salazar, 4099–001 Porto, Portugal; 4 Department of Mathematics, Faculty of Sciences, University of Porto, Rua do Campo Alegre, 4169–007 Porto, Portugal; 5 CMUP-Centre of Mathematics, University of Porto, Rua do Campo Alegre, 4169–007 Porto, Portugal; 6 Obstetrics-Gynecology, Private Hospital Trofa, 4785–409 Trofa, Portugal; 7 Gulbenkian Program for Advanced Medical Education, 1067–001 Lisbon, Portugal; 8 Department of Medicine, Beth Israel Deaconess Medical Center, Harvard Medical School, Boston, Massachusetts, United States of America; 9 Faculty of Nutrition and Food Sciences, University of Porto, Rua Dr. Roberto Frias, 4200–465 Porto, Portugal; 10 Department of Cardiology, Faculty of Medicine, University of Porto, 4200–319 Porto, Portugal; 11 Obstetrics-Gynecology, Hospital-CUF Porto, 4100–180 Porto, Portugal; University of Barcelona, SPAIN

## Abstract

**Background:**

Cyclic endometrial neoangiogenesis contributes to changes in local vascular patterns and is amenable to non-invasive assessment with Doppler sonography. We hypothesize that the uterine artery (UtA) impedance, measured by its pulsatility index (PI), exhibits a regular pattern during the normal menstrual cycle. Therefore, the main study objective was to derive normative new day-cycle-based reference ranges for the UtA-PI during the entire cycle from days 1 to 34 according to the isolated time effect and potential confounders such as age and parity.

**Methods:**

From January 2009 to December 2012, a cross-sectional study of 1,821 healthy women undergoing routine gynaecological ultrasound was performed. The Doppler flow of the right and left UtA-PI was studied transvaginally by colour and pulsed Doppler imaging. The mean right and left values and the presence or absence of a bilateral protodiastolic notch were recorded. Reference intervals for the PI according to the cycle day were generated by classical linear regression.

**Results:**

The majority of patients (97.5%) presented unilateral or bilateral UtA notches. The crude 5th, 50th, and 95th reference percentile curves of the UtA-PI at 1–34 days of the normal menstrual cycle were derived. In all curves, a progressive significant decrease occurred during the first 13 days, followed by an increase and recovery in the UtA-PI. The adjusted 5th, 50th, and 95th reference percentile curves for the effects of age and parity were also obtained. These two conditions generated an approximately identical UtA-PI pattern during the cycle, except with small but significant reductions at the temporal extremes.

**Conclusions:**

The median, 5th, and the 95th percentiles of the UtA-PI decrease during the first third of the menstrual cycle and recover to their initial values during the last two thirds of the cycle. The rates of decrease and recovery depend significantly on age and parity.

## Introduction

The uterus requires an adequate blood supply to fulfil its essential role in human reproduction. As the uterine artery (UtA) provides most of the perfusion, assessment of its vascular properties is expected to provide important information on the uterine ability to allow the fertilized ovum to implant and pregnancy to progress. Doppler ultrasound has become a mainstay in the assessment of such properties because of the development of adequate, non-invasive procedures and easy-to-use equipment. Using Doppler ultrasound, a variety of circulatory data can be estimated and integrated into a quantitative determination of different impedance parameters. Of these, major importance has been attributed to the pulsatility index (PI) because it appears to more appropriately describe the blood velocity waveform [1].

Pregnancy is the most impressive change that occurs in the uterus. The nutritionally demanding growing foetus necessitates a large and progressive adaptation in the pelvic circulation, which includes the UtA and the internal iliac artery, from where it is derived [2]. In the non-pregnant condition, the UtA Doppler waveform velocity shows a systolic flow rapid rise and sudden fall that is immediately followed by a notch during early diastole [3]; however, this high impedance feature progressively disappears during pregnancy and is present in only 5% of women from 25 weeks onwards [1]. In this context, the most important change in the UtA-PI is its progressive decrement [1,4]. This change is related to major changes at the placental bed and in the uterine artery itself, which shifts from a resistance vessel to a capacitance vessel to cope with the foetal demands. Such change is so important that, during the second trimester, the uterine artery PI increases rather than decreases, and combined with the notch presence, is considered a good predictor of preeclampsia and severe intra-uterine growth restriction [5].

The monthly cyclic changes in the non-pregnant genital tract also suggest that regulated changes occur in the uterine blood supply to endow the endometrium with the ability to receive the ovum, should fertilization occur. Indeed, such cyclic changes were the subject of a number of studies measuring circulatory data in the UtA and its distal branches such as the radial and spiral arteries. The reports, however, evidenced complex temporal relationships and sometimes conflicting results.

Both a lack of significant UtA impedance changes during the cycle [6] and higher UtA-PI early and late in the cycle, with a comparatively lower level during the mid-cycle period or luteal phase, were reported [7,8]. In addition, other reports have found small peaks at mid-cycle just before ovulation [9] that appeared to interrupt the seeming regularity of UtA-PI trends. Interestingly, studies of the UtA distal branches indicated low impedance to flow during the late follicular and midluteal phases [8,10], times when the endometrial thickness and vascularity increased [11]. The decreased UtA-PI, particularly during the luteal phase, together with the increased blood velocity in the UtA and its vascular network, indicated increased uterine perfusion in preparation for implantation [8,9]. In fact, low PI of the uterine artery [12] and endometrial flow [13] were associated with improved implantation rates, further supporting the view that increased local perfusion favours successful establishment of pregnancy.

It should be emphasized that, in contrast to the UtA distal branches, the data for the UtA itself have been less consistent. While some studies failed to evidence any association [13–15], others reported an association between the lower UtA-PI at mid-cycle, higher pregnancy rates [12,16,17], and fewer miscarriage events [18]. These findings indicate that a more in-depth knowledge of the uterine circulation during the normal menstrual cycle (NMC) will provide relevant insights on reproductive physiological changes and allow the recognition of abnormal patterns; in turn, these data would prove useful in the management of reproductive disorders such as polycystic ovary syndrome, miscarriage, and repeated abortion.

Notwithstanding the gains afforded by previous investigations in unveiling UtA impedance variations during the menstrual cycle, these studies were limited by the number of women enrolled and the duration that each cycle was evaluated. In addition, day-cycle-based reference ranges for the mean UtA-PI have not been established using well-established methodological guidelines [19–21], which would prove helpful in the management of fertility disorders such as those mentioned above.

These shortcomings led us to determine normative new reference ranges for the UtA-PI based on the day-cycle from days 1 to 34 of the NMC, while also isolating the time effect and adjusting the findings for potential confounders such as age and parity.

## Materials and Methods

### Subjects

The research protocol was approved by the ethics committee (IRB protocol number: 150–13[096-DEFI/122-CES]) of Centro Hospitalar do Porto, Unidade Maternidade Júlio Dinis (CHP-MJD), and all subjects provided written informed consent.

A cross-sectional study of 1821 healthy women undergoing routine gynaecological ultrasound examination was performed from January 2009 to December 2012. During the first appointment that coincided with the ultrasound evaluation, subjects were examined by a senior specialist who reviewed the patient’s history, and verified the absence of previous hypertension, structural heart disease, diabetes and other endocrine disorders, immune disease, renal and haematological conditions, and chronic infections. A detailed gynaecological examination ruled out the presence of any pelvic or gynaecological abnormality. Inclusion criteria were: identification of the first day of the most recent menstrual period (day 1); regular menstrual cycles; absence of gynaecological disorders, menorrhagia, and established pelvic pathology on transvaginal ultrasound examination (including fibroids, abnormal sizes or clusters of ovarian cysts, and tubal disease); no chronic medication, including hormonal contraception, for the preceding 4 months; and absence of pregnancy as confirmed by ultrasound.

On the day of ultrasound examination, a menstrual calendar was handed to the patient, and a subsequent clinical appointment was scheduled 60–90 days later. The date and duration of the most recent menstruation and the date of the ultrasound evaluation were recorded on the menstrual calendar. Additionally, patients were instructed to write the dates of subsequent menstruation. Patients failing to complete the menstrual calendar or those becoming pregnant during follow-up were excluded.

### Doppler flow assessment

The ultrasound examination was performed with the woman in the lithotomy position and at any time of day. Uterine artery Doppler evaluation was performed using a Voluson 730 Pro (GE Healthcare Technologies, Milwaukee, WI, USA) ultrasound unit containing multifrequency transvaginal and transabdominal transducers. Assessments were performed by a single operator with vast experience in Doppler ultrasound to avoid inter-observer variability using a transvaginal transducer. A sagittal image of the uterus that included the cervical canal and internal cervical os was obtained. The transducer was then gently tilted from side to side, and colour flow mapping was used to identify each uterine artery at the level of the internal os. Pulsed wave Doppler was used with a sampling gate set at 2 mm to image the entire vessel and ensure that the angle of insonation was less than 30°. UtA-PIs were measured automatically as follows:

$$PI\;=\;\frac{systolic\;peak\;velocity-end\;diastolic\;velocity}{mean\;velocity\;during\;cardiac\;cycle}$$

Three similar consecutive waveforms were obtained, and the mean PI of the left and right arteries was calculated. The presence or absence of a bilateral early protodiastolic notch in UtA was evaluated. A positive notch was defined as a persistent decrease in the blood flow velocity during early diastole that was less than the diastolic peak velocity in at least one UtA Doppler ultrasound spectrum. Absence of the notch was defined by its bilateral absence.

Intraobserver reliability was obtained from two readings performed at the beginning and end of the examination during the first 100 recordings of pulsatility indices in the uterine arteries.

### Statistical analysis

**Fig 1:**
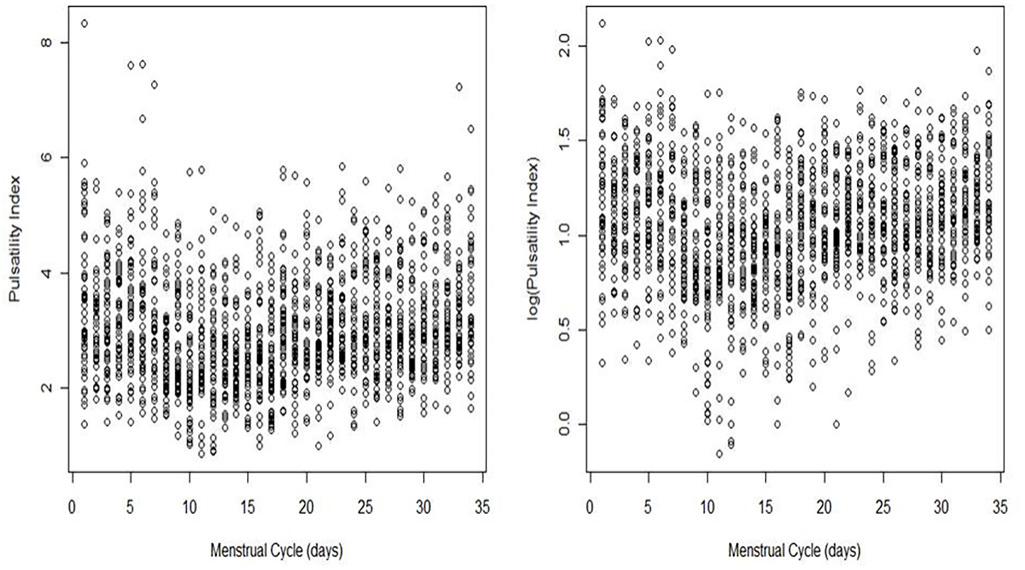
Plot of PI (left panel) and the log(PI) (right panel) measured in the uterine artery during the menstrual cycle. PI, pulsatility index.

The Chi-squared test assessed the homogeneity of proportions for categorical variables. The population reference intervals for PI were derived by regression modelling of the PI values over time during the menstrual cycle. The response was log-transformed because of the positive skewness observed in the empirical distribution (Fig. 1). Age group, Body Mass Index (BMI), parity status (primiparous vs multiparous), and smoking were considered potential time-effect confounders. However, adequate adjustment for these variables identified age and parity as the only statistically significant confounders. The crude and adjusted (for age group and parity) trends of the PI during the menstrual cycle were identified.

To study the crude effect of the menstrual cycle progression on the UtA-PI, a cubic polynomial fit the data significantly better than did a quadratic. No polynomial of a degree higher than three was considered, as those curves may exhibit unrealistic features such as waviness or sharp deviation at extreme values of the days [19]. Each day of the menstrual cycle was denoted as *d*, and a fitted model was generated as follows:

$$E(\log\left(PI\right)\;|\;d)\;=\;\beta_{0}+\beta_{1}\frac{d}{10}+\beta_{2}{\left(\frac{d}{10}\right)}^{2}+\beta_{3}{\left(\frac{d}{10}\right)}^{3}\;$$ (1)

with constants *β*_0_ *β*_1_ *β*_2_ *β*_3_ and a rescaling in the variable *d* to avoid very small regression coefficients. This equation was rewritten using multiplicative effects as follows:

$$E(PI\;|\;d)\;=\;Ce^{\gamma_{1}d+\gamma_{2}d^{2}+\gamma_{3}d^{3}}\;$$

with $C\;=\;e^{\beta_{0}},\;\gamma_{1}\;=\;\frac{\beta_{1}}{10},\;\gamma_{2}\;=\;\frac{\beta_{2}}{10^{2}}$ and $\gamma_{3}\;=\;\frac{\beta_{3}}{10^{3}}$. The letter 𝔼 in both equations denoted the conditional expected value.

Every centile curve for PI was then estimated by the following equation:

$$centile(d)\;=\;exp[\hat{\log\left(PI\right)}(d)+K\times \hat{sd})].\;$$

Here,$\log\hat{\left(PI\right)}\left(d\right)$ is the predicted response at day *d* of model (1), K is the corresponding centile of the standard Gaussian distribution, and $\hat{sd}$ is the standard deviation of the unscaled residuals of model (1).

To obtain centile curves stratified by age group (group 1, 18–26 years; group 2, 27–35 years; group 3, 36–50 years) and parity (nulliparous *vs* parous), the above regression procedure was refined. For age group *a* and parity status *p*, the best fitted model was as follows:

$$E(\log\left(PI\right)|\left(d,\;a,\;p\right))\;=\;\beta_{0}\left(a,p\right)+\beta_{1}\left(a\right)\frac{d}{10}+\beta_{2}\left(a\right){\left(\frac{d}{10}\right)}^{2}+\beta_{3}{\left(\frac{d}{10}\right)}^{3}\;$$ (2)

with *β*_0_ depending on the two considered factors, *β*_1_ and *β*_2_ depending only on the age group, and *β*_3_ designated as a constant. The reference categories corresponded to the youngest and the nulliparous classes.

Intraclass correlation coefficients (ICC) and 95% confidence intervals were calculated using a two-way mixed-effects model with absolute agreement. The reliability coefficient, which is the difference value exceeded by only 5% of pairs of measurements in a single subject, was calculated as 1.96 times the standard deviation of the difference between pairs of repeated measurements [22].

All statistical analyses were carried out using the R language and software environment for statistical computation, version 2.12.1 [23]. The significance level was fixed at 0.05.

The study adhered to the STROBE (Strengthening the Reporting of Observational studies in Epidemiology) guidelines for observational studies, and all recommendations were included in the study [S1 Table].

## Results

A total of 1821 healthy women were considered eligible for this study. Of these, 153 were excluded (8.4%); 128 women did not have clinical records in the menstrual calendar according to the study protocol; 11 women were pregnant at the time of ultrasound assessment; in 10 cases, the pulsatility index in the uterine arteries could not be measured because of technical difficulties; and four women refused to participate in the study.

The demographic characteristics of the 1668 women included in the study are summarized in Table 1. Their ages ranged from 18 to 50 years old, and 41.4% were older than 35 years. Additionally, 39.1% were nulliparous, and the majority of the patients (97.5%) exhibited a notch in the uterine arteries (unilaterally or bilaterally).

**Table 1:** Demographic characteristics of the 1668 women included in the study.

		n(%)
Age (intervals in years)	Group 1. 18–26	251 (15.0)
	Group 2. 27–35	727 (43.6)
	Group 3. 36–50	690 (41.4)
Body Mass Index^a^ (Kg/m^2^)	16–24	1032 (61.9)
	25–29	480 (28.8)
	30–39	156 (9.3)
Parity	0	653 (39.1)
	≥1	1015 (60.9)
Age at menarche, years (mean±SD)	12.1 (1.17)	-
Age at first sexual intercourse (years±SD)	17.9 (2.33)	-
History of miscarriage	No	1479 (88.7)
	Yes	188 (11.3)
History of preeclampsia	No	1644 (98.6)
	Yes	24 (1.4)
Smoking	No	1380 (82.7)
	Yes	288 (17.3)
Presence of bilateral notching	No	100 (6.0)
	Yes	1568 (94.0)
Presence of unilateral notching	No	42(2.5)
	Yes	1626 (97.5)
Menstrual cycle length, days (mean±SD)	28.8(4.2)	-
Menstrual period length, days (mean±SD)	5.0(1.7)	-

SD, standard deviation

^a^Body Mass Index (BMI) was measured immediately before Doppler assessment.

### UtA-PI during the normal menstrual cycle

The reliability coefficient for the UtA-PI was 0.434. The ICC for the intraobserver reliability of the UtA-PI measurements was 0.984, with a 95% confidence interval ranging from 0.976 to 0.989.

The days evaluated in the menstrual cycle varied from 1 to 34, and the collected data were slightly unbalanced. The least frequently assessed point was day 34 (39 patients), and the most frequently assessed were days 22 and 26 (54 patients each) (Table 2). However, the empirical distribution for the day number was essentially uniform, with a sample mean ± standard deviation of 17.3 ± 9.7 compared with 17.0 ± 9.8 expected in a uniform distribution.

**Table 2:** Observed and predicted percentiles of the uterine artery pulsatility index on each cycle day.

			Observed			Predicted		
Cycle (days)	n	EOD^a^ (n)	5^th^ centile	50^th^ centile	95^th^ centile	5^th^ centile	50^th^ centile	95^th^ centile
1	50	0	1.74	3.37	5.55	2.08	3.40	5.55
2	50	0	2.00	3.04	4.59	1.99	3.24	5.29
3	49	0	1.88	3.02	4.90	1.90	3.11	5.07
4	48	0	1.83	3.17	4.60	1.83	2.99	4.89
5	51	0	1.94	3.25	5.00	1.78	2.90	4.73
6	52	1	1.88	3.15	5.77	1.73	2.82	4.60
7	46	20	1.80	3.12	5.37	1.69	2.76	4.50
8	52	46	1.59	2.41	4.03	1.66	2.71	4.42
9	50	107	1.83	2.48	4.64	1.63	2.67	4.36
10	52	110	1.09	2.21	3.93	1.62	2.64	4.31
11	49	136	1.24	2.40	4.48	1.61	2.62	4.28
12	48	131	1.05	2.60	4.10	1.60	2.61	4.27
13	49	103	1.52	2.62	4.18	1.60	2.61	4.26
14	53	209	1.81	2.33	4.09	1.60	2.62	4.27
15	46	160	1.42	2.56	3.66	1.61	2.63	4.29
16	50	71	1.43	2.60	4.87	1.62	2.65	4.32
17	52	104	1.36	2.33	3.93	1.63	2.67	4.36
18	52	103	1.93	2.99	4.66	1.65	2.70	4.40
19	49	67	1.62	2.88	3.96	1.67	2.73	4.45
20	49	99	1.89	2.74	4.78	1.69	2.76	4.51
21	50	96	1.85	2.72	4.31	1.72	2.80	4.57
22	54	61	2.14	3.01	4.18	1.74	2.84	4.64
23	48	19	2.30	2.86	5.03	1.77	2.88	4.70
24	45	21	1.56	2.91	4.81	1.79	2.92	4.77
25	51	4	1.81	3.07	4.73	1.82	2.97	4.84
26	54	0	1.87	2.96	4.30	1.84	3.01	4.91
27	46	0	2.08	2.86	4.71	1.87	3.05	4.98
28	47	0	1.67	2.94	4.52	1.89	3.09	5.04
29	47	0	2.16	2.90	4.58	1.91	3.12	5.09
30	50	0	1.97	3.00	4.61	1.93	3.15	5.14
31	43	0	2.19	2.94	4.90	1.94	3.17	5.18
32	49	0	2.16	3.25	4.95	1.95	3.19	5.21
33	48	0	2.41	3.14	4.29	1.96	3.20	5.23
34	39	0	2.11	3.21	5.42	1.96	3.20	5.23

^a^For each patient, the expected ovulation date (EOD) was calculated assuming that the luteal phase exhibited a consistent duration of approximately 2 weeks, i.e., *E0D* = *menstrual cycle length*—14.

**Fig 2:**
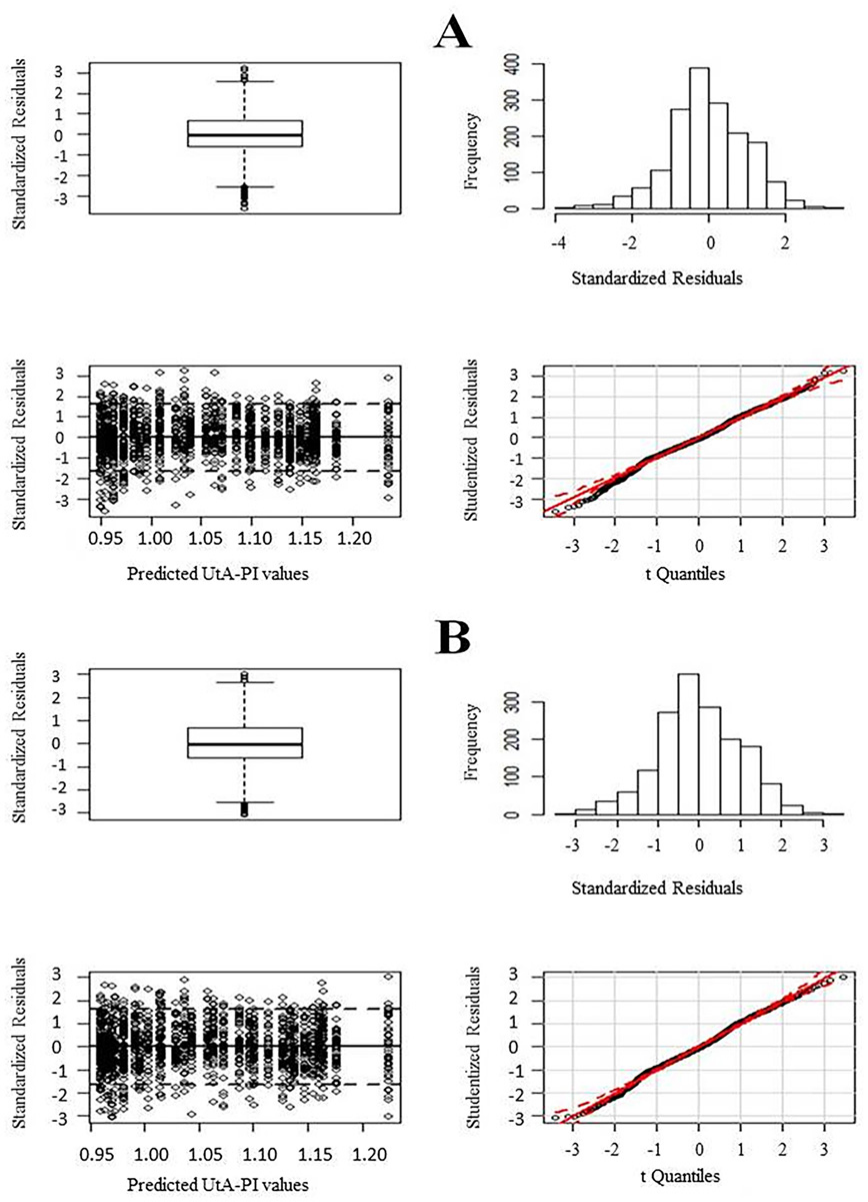
Residual plots for the fitted models; from top to bottom and from left to right: boxplot of the standardized residuals, histogram of the standardized residuals, plot of the standardized residuals against the predicted values, and QQ-plot of the studentized residuals. Panel A employs the entire dataset; Panel B eliminates data from 11 outliers.

Concerning the fitting of model (1), visual inspection of the normality and homoscedasticity of the residuals was performed (Fig. 2, Panel A). There were no serious departures from normality except at a few extreme points, mostly located on the left tail. Data with an absolute value of the standardized residuals greater than three were removed. A total of 11 data points were removed (eight on the left tail), corresponding to less than 1% of the total sample size. The highest and lowest cutoff values for PI in these women were 7.26 and 1.03, respectively.

All parameter estimates of the final fitted model were statistically significant (Table 3). Standard errors of the estimates were up to 3% smaller than the errors in the model using the total data. Residual plots exhibited reasonable properties for normality adherence (Fig. 2, Panel B): 89% of the standardized residuals lay between-1.645 and 1.645; the boxplot revealed an approximately symmetric distribution with the median line at approximately the centre of the box and symmetric whiskers; and the quantile-quantile (Q-Q) plot of the studentized residuals showed little departure from the confidence band for the correspondent *t* distribution. In addition, the Lilliefors-corrected Kolmogorov-Smirnov normality test provided a p-value of 0.002; this statistical significance was overlooked because of the large sample size (n = 1657). The outliers were again removed, but the results were no better.

**Table 3:** Estimates of the regression coefficients and corresponding 95% confidence intervals (CI) of model (1) fitted without the 11 identified outliers.

Variables	Regression Coefficients	95% CI
Intercept	1.279	(1.214, 1.344)*
Day/10	-0.573	(-0.730, -0.416)*
(Day/10)^2^	0.310	(0.206, 0.414)*
(Day/10)^2^	-0.044	(-0.064, -0.024)*

*Significant at the 0.05 level.

The plot of the logarithmized UtA-PI values against the days of the cycle did not show any substantive changes in the standard deviations of the values along the menstrual cycle (Fig. 1); however, a formal statistical model for this relationship was applied. The linear regression of the scaled absolute residuals (SARs), defined as the product of $\sqrt{\pi /2}$ by the absolute residuals, on a polynomial of degree 1 in the variable *Day* was statistically significant (p < 0.001), and no higher-order terms were identified. As this regression only explained approximately 1% of the SARs’ total variability, it was considered redundant, and therefore the residual homoscedasticity of model (1) did not appear violated.

**Fig 3:**
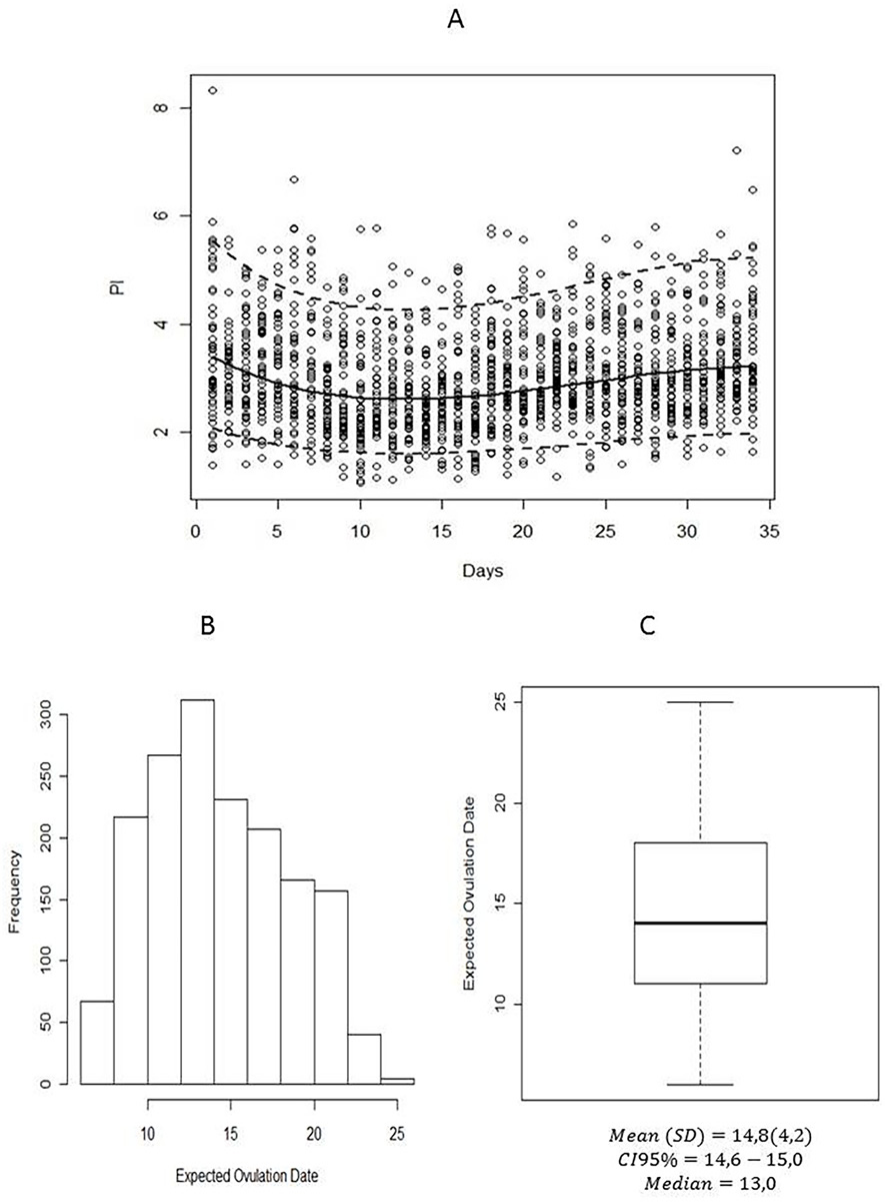
(A) Observed (circles) and predicted 5^th^, 50^th^, and 95^th^ percentile regression curves for the pulsatility index during the menstrual cycle. Histogram (B) and boxplot (C) of the expected ovulation date (EOD). For each patient, the EOD was calculated assuming that the luteal phase exhibited a consistent duration of approximately 2 weeks, i.e., *E0D = menstrual cycle length—*14.

The predicted 5^th^, 50^th^, and 95^th^ percentile regression curves are presented in Table 2 and plotted in Fig. 3. The expected ovulation date (EOD) was calculated in each patient assuming that the luteal phase lasted approximately 2 weeks (*i*.*e*., *E0D = menstrual cycle length—*14). Accordingly, the 50^th^ centile curve for PI, which under the normality assumption coincides with the mean curve, began at day 1 at its maximum value (3.40) and decreased until reaching its minimum value at day 12–13. From this day onwards, the curve increased until reaching 3.20 (50^th^ centile) at the end of the menstrual cycle (day 34). If the curve failed to stop at day 34, it continued to decrease afterwards. Day 34 corresponded to the local maximum of the defined function.

As the 5^th^ and the 95^th^ centile curves were simply the product of the 50^th^ percentile and a constant value, they showed the same monotonicity behaviour as the 50^th^ centile curve, with maximum and minimum values attained on the same days (Table 2). The 5^th^ centile curve began at 2.08, ended at a similar PI value of 1.96, and had a minimum PI value of 1.60. The 95^th^ centile curve began at 5.55, ended at 5.23, and had a minimum PI value of 4.27. The steepest decrease in the PI during the first 12–13 days of the menstrual cycle occurred in the 95^th^ centile curve, and the smallest decrease occurred in the 5^th^ centile curve (Fig. 3).

### Effect of maternal age and parity on UtA-PI during normal menstrual cycle

To obtain centile curves stratified by age group and parity status, the regression procedure was refined taking these factors into consideration, while also excluding the same 11 data points as previously. The best fitted model is described in model (2), and the estimated regression coefficients and corresponding 95% confidence intervals are summarized in Table 4. All estimates but one (a quadratic term on days of the menstrual cycle for women aged 27–35 years, p = 0.089) were statistically significant.

**Table 4:** Estimates of the regression coefficients and corresponding 95% confidence intervals (CI) for the model stratified by age and parity.

Variables	Regression Coefficients	95% CI
Intercept	1.546	(1.438, 1.654)*
Age Group 2	-0.236	(-0.361, -0.110)*
Age Group 3	-0.335	(-0.463, -0.206)*
Parous	-0.066	(-0.098, -0.034)*
Day/10	-0.792	(-0.992, -0.592)*
(Day/10)^2^	0.353	(0.244, 0.461)*
(Day/10)^3^	-0.042	(-0.062, -0.023)*
(Age Group 2):(Day/10)	0.216	(0.041, 0.391)*
(Age Group 3):(Day/10)	0.365	(0.188, 0.543)*
(Age Group 2):(Day/10)^2^	-0.044	(-0.044, 0.026)
(Age Group 3):(Day/10)^2^	-0.091	(-0.142, -0.039)*

*Significant at the 0.05 level.

**Fig 4:**
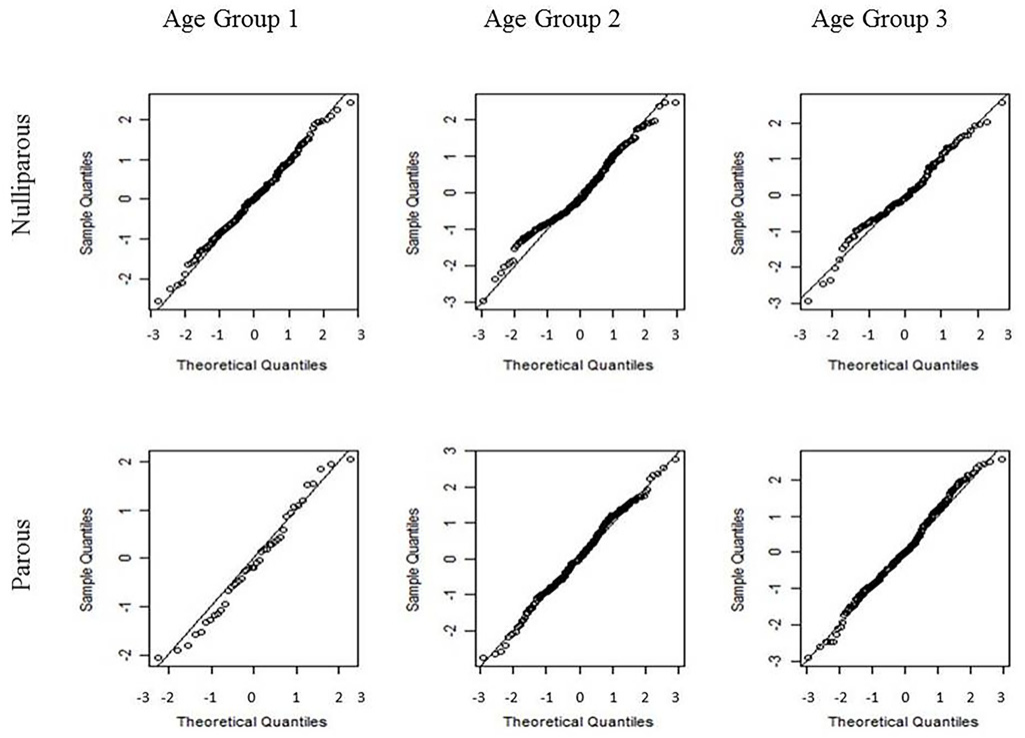
Quantile-quantile (Q-Q) plots of the standardized residuals of model (2) for each combination of age and parity groups. Nulliparous women are presented in the first row, and parous women are presented in the second row. The age group increases from the left to right columns. From left to right and from top to bottom, the Lilliefors-corrected Kolmogorov-Smirnov normality test calculated p-values of 0.738, <0.001, 0.111, 0.954, 0.082, and 0.373, respectively.

**Fig 5:**
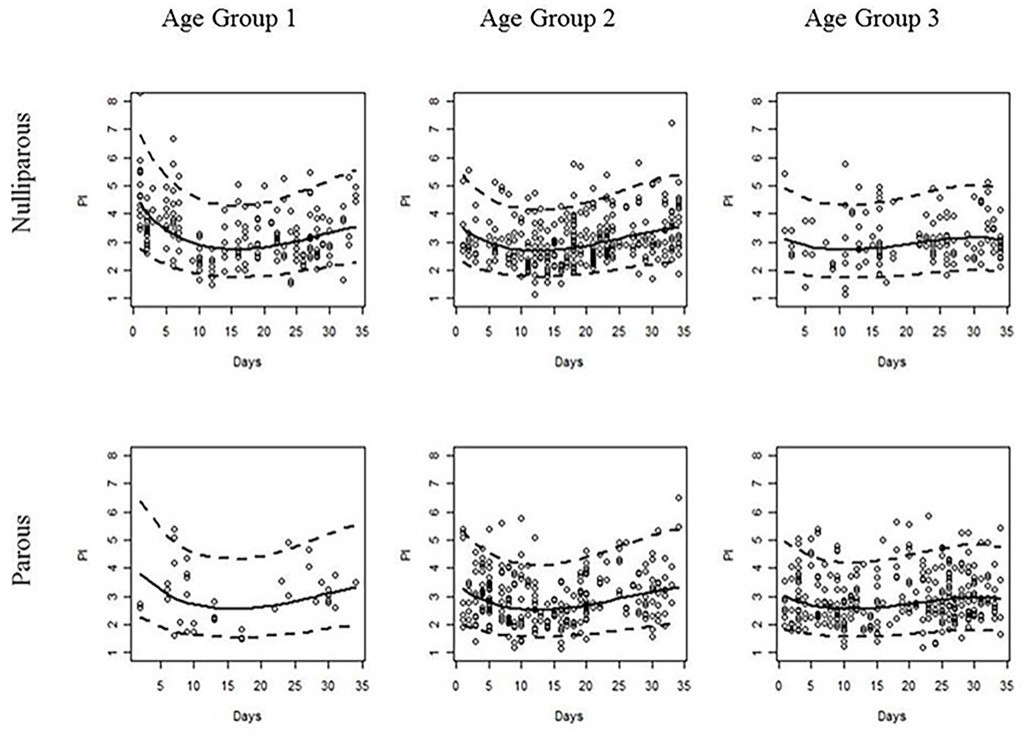
Observed (points) and estimated 5^th^, 50^th^, and 95^th^ percentile regression curves of the pulsatility index during the menstrual cycle for each combination of age and parity groups. Nulliparous and parous women are presented in the first and second rows, respectively. The age group increases from the left to right columns.

No significant interaction effects involving parity were identified. No serious outliers or evidence of violations in normality and homoscedasticity assumptions were detected within each age-parity sub-model (Figs. 4 and 5). The residual regression on the explanatory variables, with a linear dependence on the cycle days, presented a value for the coefficient of determination (*R* ^2^) of approximately 1% and did not identify the effect of age group as statistically significant. As before, the residual homoscedasticity of the model did not appear compromised. The residual normality could only be rejected for nulliparous women aged 27–35 years (Fig. 4).

The standard deviation of the residuals of model (2) did not significantly change during the menstrual cycle; therefore, the coefficient significance in Table 4 remained true for all centile curves.

For any fixed age group and centile curve, a significant difference between the intercepts of the curves for nulliparous and parous women was identified, with the menstrual cycle in nulliparous women beginning at higher UtA-PI values. Similarly, for any fixed parity status and centile curve, there were significant differences between the intercept of the age group 2 (or 3) curve and that of age group 1 (Fig. 5).

Overall, the PI values exhibited a soft wave-like trend during the menstrual cycle within each maternal age and parity group. The values decreased until approximately the first third of the menstrual cycle and then increased to approximately the original value over the remainder of the cycle. This trend was independent from the parity status but was significantly dependent on the maternal age at the linear and quadratic levels. As the age increased, the minimum UtA-PI was reached more quickly, the range of the PI values decreased, and the curves became flatter (Fig. 5).

## Discussion

Transvaginal assessment of UtA perfusion employing Doppler ultrasound offers several advantages over the transabdominal route [24]. The vessel is easily identified and located at close proximity, thus yielding clearer waveforms, and the insonation angle is near 0°, which results in high reproducibility [24,25]. Despite the diversity of features that may be determined through arterial resistance, it is impedance, the combination of forward and reflected blood flow, that is measured [26]. This is accomplished by indirectly measuring the UtA-PI, a technique that has gained popularity in recent studies compared with other Doppler indices such as resistance index [1,27] and other scoring systems [28,29].

These principles were applied in the current study to generate Doppler colour-based reference ranges for the mean UtA-PI between days 1 and 34 of the NMC in an appropriately large sample of healthy women. In addition, the effects of age and parity were assessed for each day during the NMC.

### Statistics

The reliability evaluation demonstrated that UtA-PI measurement was highly repeatable as indicated by the ICC. There is sufficient scientific consensus that an ICC > 0.7 reflects very low measurement error [22,30].

Stringer and validated methodological guidelines were used to construct the reference curves from the collected data [19–21,31]; a cross-sectional design was used as such studies are easier to perform and combine with clinical practice; and finally, the good intraobserver reproducibility in our study suggests that the mean UtA-PI is a reliable parameter in a clinical setting. The overarching principle is that a reference interval is the range of values encompassed by a pair of symmetrically placed extreme centiles, such as the 2.5^th^ and 97.5^th^ centiles for a 95% interval [19]. Values lying outside the reference limits are considered unusual or extreme.

Several statistical methods have been used to generate reference intervals: linear regression (if necessary with modelling of the residual standard deviation), the LMS method [32], the non-parametric method of Healy, Rabash, and Young (HRY), and non-parametric quantile regression [21]. Each method has its advantages and limitations; however, the method that is most desired is the one allowing identification of the population centile of a given observation. Linear regression has that property and is simple and easily implemented by basic statistical software packages.

### Perfusion changes during the normal menstrual cycle

The current study revealed a cyclic variation in the UtA impedance during the NMC; the UtA-PI was high during the temporal extremes and showed a mid-cycle depression, with the minimal values occurring between days 13 and 17. Independent assessment of the effects of age and parity also revealed that both conditions were associated with a significant decrease in the UtA impedance at the extremes of the cycle, but not during the mid-cycle, when the uterus undergoes impressive structural changes. These circulatory variations include the median, 5^th^, and 95^th^ percentiles of the UtA-PI regression curves from the initial through to the final third of the menstrual cycle. The mechanism underlying such impedance variation is unknown, but it likely reflects regulatory factors affecting the local vasculature and myometrium function.

Early during the cycle, increased myometrial tone is required to expel the remains of the sloughing endometrium; for this purpose, the smooth muscle cells contract, which has a negative effect on uterine perfusion and generates high impedance to UtA blood flow. We suspect that a similar muscular change underlies the UtA-PI rise near the end of the cycle.

There is evidence that variations in muscular tone reflect circulating levels of female steroid hormones, particularly oestrogen, which is low early in the cycle but increases later during the follicular phase. Oestrogen promotes vascular smooth muscle relaxation and reduces sensitivity to adrenergic stimulation [33]; moreover, in experimental conditions, it was found to depress uterine contractility both *in vivo* [34] and in freshly isolated rat uterine specimens [35]. This decrease in myometrial tone and increasingly thickened endometrium during the proliferative phase, together with the development of an extensive small vessel network, is the likely cause for the downward trend in impedance that reaches a minimum near day 13. After ovulation, progesterone concentration rises through the mid-secretory phase, promoting endometrium decidualization. Oestrogen decrement [36] and the ability of progesterone to overcome the inhibitory action of 17β-oestradiol on smooth muscle contractility [34,35] favour the rise in UtA-PI.

Other important molecules, acting independently or under the effect of sex steroids, may contribute to the circulatory changes. Prostanoids such as prostaglandin F_2α_ and latanoprost promote murine [35,36] and human [35] myometrial contractility, but their blood concentration variation during the cycle is uncertain. In addition, vasopressin and oxytocin stimulate uterine contraction via myometrial vasopressin V_1a_ and oxytocin receptors [37]. Near the end of the cycle, although circulating at a lower concentration [38], vasopressin exerts a stronger effect than oxytocin [37]. Therefore, although the known actions of these compounds on the myometrium and its perfusion are appealing, their role during the cycle remains to be established.

### Effect of parity and age on uterine flow impedance

In the current study, in non-pregnant women, the UtA-PI early and late in the cycle was significantly lower in parous women than in nulliparous women. This point has not been examined in any known reports previously.

UtA-PI reduction is important because it is accompanied by improved myometrium perfusion, which provides local benefits. Indeed, it has been suggested that impaired uterine perfusion is a cause for unexplained infertility [39] and is reportedly a predictive indicator for the implantation and pregnancy outcomes [40–42]. Moreover, upon pregnancy establishment, parous women appear to have improved perfusion. In fact, as early as the first trimester, parous women exhibited lower UtA-PI and total peripheral resistance compared with those in nulliparous women [43], a finding that was also described in twin pregnancies compared between parous and nulliparous women [44]. Furthermore, all reports indicate that parous women have a lower prevalence of protodiastolic notching [45,46], a feature whose persistence is associated with a poor prognosis [5].

The enhanced perfusion in parous women likely results from vascular structural features that persist after the first pregnancy. Shortly after implantation, the spiral arteries undergo remarkable structural remodelling, which is necessary to accommodate the increased uteroplacental perfusion [47]. At the end of pregnancy, these largely regress, but not entirely; in contrast to nulliparous women, spiral artery internal elastic lamina duplication or fragmentation has been observed at the endometrial/myometrial junction of parous women [48]. Such permanent structural changes endow spiral arteries with reduced impedance that supports the parity-related UtA-PI reduction here reported.

Similar to parous women, the UtA-PI decreased at the extremes of the cycle in older women when compared with younger women. Interestingly, uterine stripes from aged non-pregnant women exhibited reduced contractility either spontaneously or upon exposure to oxytocin [49], suggesting that reduced uterine muscular tone underlies the lessened UtA impedance. The cause for this sluggish response in unknown but may be consequent to an age-related change in local regulation. For example, in the pregnant uterus, the UtA-PI shows a general decreasing trend starting at the first trimester [1,2,4]; yet, when the UtA is measured at specific pregnancy time-points, a slight age-related increase may be observed [50,51]. Degenerative changes in the UtA wall, present even before menopause [52], or other local factors are the likely contributors to this particular observation.

Therefore, both age and parity similarly affect the UtA-PI of non-pregnant women during the NMC. Interestingly, the downward trend favours perfusion, which appears to bear reproductive benefits. Infants born to parous women tend to have increased birth weight [43], while older pregnant women tend to have increased placental weights [53], but the significance of these trends remains undefined.

### Study limitations and future research

(1) The study was conducted in a sample of healthy women. (2) Further studies are necessary to assess abnormal uterine artery PI as a diagnostic or prognostic tool of reproductive disorders as the ovulation day was not identified, and the endometrial structure was not examined. (3) Our data were collected by a single, experienced operator, which could compromise the external validity of his results. Because the usefulness of a screening test depends not only on its predictive ability but also on its reproducibility, future studies are needed to demonstrate the usefulness of these reference ranges, as well as their applicability.

## Conclusions

This cross-sectional study employing a large set of women evidences a clear decrement in the UtA impedance during the middle of the menstrual cycle compared with its extremities. For unknown reasons, age and parity do not change this trend; they instead flatten the extremes while leaving the mid-cycle unchanged, suggesting that local mechanisms regulate an adequate uterine perfusion in preparation for implantation.

To the well-known cyclic structural features occurring in the uterus, the current study adds another cyclic circulatory event. The elucidation of the mechanisms underlying these changes, apart from providing new insights into the fascinating implantation process, may improve prediction of reproductive and pregnancy disorders, thus enhancing the importance of UtA assessment.

## Supporting Information

S1 Table STROBE Statement—checklist of items that should be included in reports of observational studies. (DOCX)
